# Development and Validation of a UPLC-MS/MS Method for Therapeutic Drug Monitoring, Pharmacokinetic and Stability Studies of First-Line Antituberculosis Drugs in Urine

**DOI:** 10.3390/molecules29020337

**Published:** 2024-01-09

**Authors:** Mohamed Abouzid, Katarzyna Kosicka-Noworzyń, Marta Karaźniewicz-Łada, Prakruti Rao, Nisha Modi, Yingda L. Xie, Scott K. Heysell, Anna Główka, Leonid Kagan

**Affiliations:** 1 Department of Physical Pharmacy and Pharmacokinetics, Poznan University of Medical Sciences, 3 Rokietnicka Street, 60-806 Poznań, Poland; mmahmoud@ump.edu.pl (M.A.); kasiakosicka@ump.edu.pl (K.K.-N.); 2 Doctoral School, Poznan University of Medical Sciences, 70 Bukowska Street, 60-812 Poznań, Poland; 3 Department of Pharmaceutics, Ernest Mario School of Pharmacy, Rutgers, The State University of New Jersey, 160 Frelinghuysen Road, Piscataway, NJ 08854, USA; 4 Division of Infectious Diseases and International Health, University of Virginia, 345 Crispell Drive, Charlottesville, VA 22903, USA; pr6zu@virginia.edu (P.R.); skh8r@virginia.edu (S.K.H.); 5 Public Health Research Institute, Department of Medicine, Rutgers New Jersey Medical School, Newark, NJ 07013, USA; nisha.modi@rutgers.edu (N.M.); ylx1@njms.rutgers.edu (Y.L.X.); 6 Department of Bromatology, Poznan University of Medical Sciences, 3 Rokietnicka Street, 60-806 Poznań, Poland; aglowka@ump.edu.pl; 7 Center of Excellence for Pharmaceutical Translational Research and Education, Ernest Mario School of Pharmacy, Rutgers, The State University of New Jersey, 160 Frelinghuysen Road, Piscataway, NJ 08854, USA

**Keywords:** therapeutic drug monitoring, rifampicin, isoniazid, pyrazinamide, ethambutol

## Abstract

Tuberculosis (TB) remains one of the leading global causes of mortality. Several methods have been established to detect anti-TB agents in human plasma and serum. However, there is a notable absence of studies analyzing TB drugs in urine. Thus, our objective was to validate a method for quantifying first-line anti-TB agents: isoniazid (INH), pyrazinamide (PZA), ethambutol (ETH), and rifampicin (RIF), along with its metabolite 25-desacetylrifampicin, and degradation products: rifampicin quinone and 3-formyl-rifampicin in 10 µL of urine. Chromatographic separation was achieved using a Kinetex Polar C18 analytical column with gradient elution (5 mM ammonium acetate and acetonitrile with 0.1% formic acid). Mass spectrometry detection was carried out using a triple-quadrupole tandem mass spectrometer operating in positive ion mode. The lower limit of quantification (LLOQ) was 0.5 µg/mL for INH, PZA, ETH, and RIF, and 0.1 µg/mL for RIF’s metabolites and degradation products. The method was validated following FDA guidance criteria and successfully applied to the analysis of the studied compounds in urine of TB patients. Additionally, we conducted a stability study of the anti-TB agents under various pH and temperature conditions to mimic the urine collection process in different settings (peripheral clinics or central laboratories).

## 1. Introduction

The WHO Global Tuberculosis Report has affirmed that tuberculosis (TB) is a major cause of illness and one of the leading causes of death worldwide. Prior to the coronavirus (COVID-19) pandemic, TB stood as the principal cause of death attributed to a singular infectious agent, surpassing even the impact of HIV/AIDS [1].

In 2021, global TB incidence experienced a significant increase, with an estimated 10.6 million individuals becoming ill, representing a 4.5% rise from the 10.1 million cases reported in 2020 [1]. Standard TB treatment recommendations involve a course lasting 4 to 6 months, utilizing anti-TB drugs and resulting in a cure rate of approximately 85%. For drug-susceptible TB, the most common treatment approach involves a 2-month intensive phase characterized by the administration of four key anti-TB drugs: rifampicin (RIF), isoniazid (INH), pyrazinamide (PZA), and ethambutol (ETH). Subsequently, a 4-month continuation phase ensues, which includes the use of INH and RIF [2].

Several papers highlighted the importance of therapeutic drug monitoring (TDM) for TB drugs [3,4,5,6]. For instance, it was observed that the genetic polymorphism of *SLCO1B1* [7], and *NAT2* [8] may reduce RIF exposure and INH-induced liver injury, respectively [7,8]. Moreover, TDM is beneficial for ensuring the safety and effectiveness of TB treatment in patients with other health conditions like diabetes, malnourishment, HIV, or kidney and liver problems that are prone to malabsorption or alterations in drug metabolism or excretion [6,9,10,11]. Traditionally, serum or plasma are matrices of choice for TDM. However, blood collecting, processing (centrifuging and pipetting), and plasma/serum shipping may demand more laboratory use and are therefore challenging, especially in high-TB burdened countries [12].

Urine testing may allow a simpler sample collection and acceptability in supporting optimal drug pharmacokinetics, adherence, and use in understanding the performance of new drug regimens in diverse communities [12,13,14,15,16,17,18]. Although urine assays encounter greater challenges than plasma assays, largely owing to nonspecific binding and variations in urine pH and salt concentration [19], urine holds numerous advantages as a matrix for pharmacokinetics and TDM. These include noninvasiveness, convenient collection, the potential for ample quantities, straightforward storage, ease of handling (no need to centrifuge and pipet), and transportation, ultimately saving valuable time for medical personnel and enhancing the overall biomedical analysis process, especially in low-resourced countries [20]. Approximately 30% of RIF dose is excreted in the urine, of which approximately half is unchanged RIF [21] compared to only 7–29% of INH [22]; 3.2% of PZA is excreted in unchanged form in the urine [23]. ETH is mainly eliminated by renal excretion in unchanged form [24]. However, several procedures were reported to quantify anti-TB agents in human plasma and serum [25], while only a few authors analyzed these drugs in urine [26,27,28,29].

To optimize routine clinical protocols for TDM in urine, key knowledge gaps around the stability of anti-TB agents must be addressed. INH demonstrates ambient room temperature instability within whole blood, serum, and plasma [4]. Current protocols indicate that specimens should be promptly frozen after collection to avoid deterioration; however, in some settings, it is challenging to freeze specimens promptly and keep them frozen while transported [4]. Furthermore, it is worth noting that RIF undergoes nonenzymatic autooxidation, leading to the formation of rifampicin quinone (RIF-Q), such as in RIF tablets, indicating impurity [30,31,32]. In acidic pH, RIF is degraded to 3-formyl-rifampicin (3-F-RIF) [33,34], which becomes more conspicuous in the presence of INH [34]. Shifting the focus to alkaline pH conditions, RIF experiences deacetylation, resulting in the emergence of 25-desacetylrifampicin (25-D-RIF), a predominant metabolite within human systems. This compound retains approximately 20% of RIF’s antimicrobial potency [35,36]. It is noteworthy that mild alkaline environments also foster RIF-Q generation [36]. In plasma, INH, PZA, RIF, and 25-D-RIF stability were discussed earlier [37]. However, there is rare information about the stability in different pH ranges, particularly in urine.

Within the scope of this investigation, a robust UPLC-MS/MS methodology was developed and validated based on FDA guidelines to facilitate the TDM of primary first-line anti-TB agents, specifically INH, PZA, RIF, and ETH in urine. Notably, this method can thoroughly analyze RIF’s stability and metabolic pathways by measuring 25-D-RIF, 3-F-RIF, and RIF-Q. To our knowledge, this is the first study of anti-TB drugs analysis in urine that evaluated stability in urine under conditions that might be encountered in a warm climate. Furthermore, the preparatory phase is both rapid and uncomplicated.

## 2. Results

### 2.1. Method Validation

#### 2.1.1. Selectivity

In detecting RIF, RIF-Q, 3-F-RIF, 25-D-RIF, INH, PZA, and ETH using MRM mode, we ensured a high level of selectivity. There were no interferences from endogenous compounds observed at the expected retention times of the analytes in blank urine samples collected from six different individuals. However, when comparing the zero urine samples to the quality control (QC) at the lower limit of quantification (LLOQ) level for these analytes, we observed some contamination of PZA-IS with PZA.

**Figure 1:**
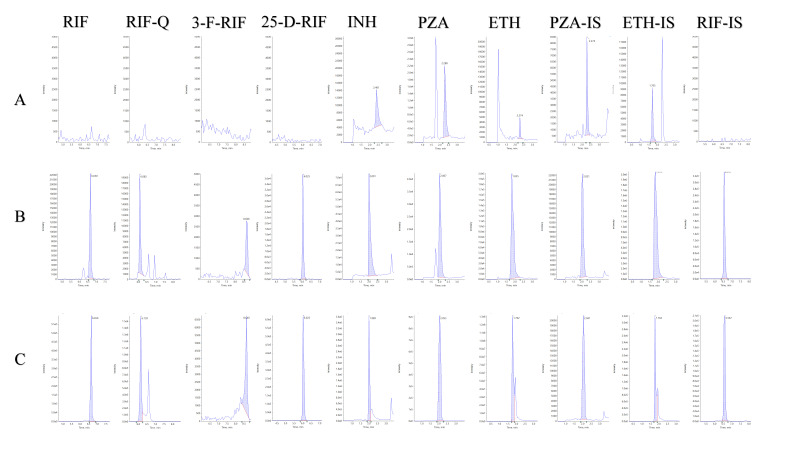
MRM chromatograms of anti-TB drugs in human urine: (**A**) unspiked blank human urine; (**B**) blank human urine spiked at the LLOQ level (0.1 mg/L for RIF, RIF-Q, 3-F-RIF, and 25-D-RIF; 0.5 mg/L for INH, PZA, RIF, and ETH), internal standards at levels 0.05 mg/L for RIF-IS, 0.5 mg/L for PZA-IS and ETH-IS; (**C**) human urine from the patient undergoing treatment for active TB disease with first-line RIF-containing regimen (measured concentrations: 18.5 mg/L for RIF, 2.4 mg/mL for RIF-Q, 0.5 mg/mL for 3-F-RIF, 3.1 mg/L for 25-D-RIF, 36.5 mg/L for INH, 79.5 mg/L for PZA, and 235.6 mg/L for ETH). Abbreviations: 25-D-RIF—25-desacetylrifampicin; 3-F-RIF—3-formylrifampicin; ETH—ethambutol; ETH-IS—ethambutol-D_4_; INH—isoniazid; PZA—pyrazinamide; PZA-IS—pyrazinamide-15N,D_3_; RIF-Q—rifampicin quinone; RIF—rifampicin; RIF-IS—rifampicin-D_3_.

Nevertheless, the signal intensity of the analyte in these zero samples was less than 20% of the signal intensity at the LLOQ level. To visually illustrate this, Figure 1 shows representative MRM chromatograms of a blank urine sample, a calibration standard containing analytes at LLOQ, and a patient sample.

#### 2.1.2. Calibration Curves

The calibration curves for 3-F-RIF, 25-D-RIF, RIF-Q, INH, PZA, RIF, and ETH were constructed with eight-point levels. These concentration ranges for the TB drugs were determined based on the anticipated urine concentrations in patients following a standard dosage regimen. The linearity of the analytical response within these calibration ranges was verified for RIF, 3-F-RIF, 25-D-RIF, INH, PZA, and ETH. This verification was achieved by applying specific weighting factors, such as 1/x^2^ (INH) or 1/x (RIF, 3-F-RIF, 25-D-RIF, PZA, and ETH). Notably, a power function provided the best fit for RIF-Q. Moreover, it was possible to extend the range of the ETH calibration curve to include 500 and 1000 µg/mL due to the higher values of ETH found in patients’ urine samples, and the calibration curve remained linear with R^2^ = 0.994 and 0.98, respectively—the same weighting also was used (1/x). The process was crucial to ensure the sample could be processed directly without dilution.

#### 2.1.3. LLOQ, Precision, and Accuracy

LLOQ intraday precision and accuracy were 6.0–19.4% and 94.3–115.7%, respectively. At the same time, the LLOQ inter-day precision and accuracy were 4.7–12.9% and 86.4–106.2%, respectively (Table 1).

For the analytes prepared at different QC levels, the intraday precision and accuracy in five replicates were 3.9–13.3% and 87.7–108.6%, respectively (Table 1), while their inter-day precision and accuracy from 5 consecutive days ranged from 1.4% to 14.3% and 90.0% to 114.0%, respectively (Table 1).

#### 2.1.4. Carry-Over

There was a significant carry-over for INH and PZA (83% and 107% of the peak’s area for LLOQ level, respectively), which was reduced to 32% and 69% of the peak’s area for LLOQ level, respectively, by implementing the cleaning procedure (Section 4.2) and one injection of a mixture of isopropanol and water (50:50, *v*/*v*). Two injections of the mixture were allowed to eliminate carry-over.

#### 2.1.5. Matrix Effect

The IS-normalized matrix factor (MF) for ETH was 0.85 and 0.83 for low and high concentrations, respectively (Table 2), indicating a minimal contribution of the matrix to ion suppression. However, the significant matrix effect was noticed for RIF-Q, INH, PZA, RIF, 3-F-RIF, and 25-D-RIF. Moreover, the analytical signal of RIF-Q, INH, and PZA varied depending on the matrix source as supported by the %RSD calculated at low and high concentrations for RIF-Q (45.2%, 51.9%), INH (12.6%, 18.3%), and PZA (16.1%, 21.7%) from six different urine sources. It is worth noting that the matrix effect assessment was repeated twice, yet similar results were obtained.

#### 2.1.6. Stability

Table 3 presents the obtained results of the analytes’ stability in urine at various storage conditions. The studied compounds (except RIF-Q) remained unchanged in urine samples at room temperature up to 1 h (percentage of nominal concentration: 87.3–112.2%).

When stored at room temperature for 4 h, the RIF-Q accuracy reduced to 38.7% and 78.7% for low and high concentrations (LQC and HQC), respectively, and the accuracy of 3-F-RIF low concentrations decreased to 61%. The accuracy of 25-D-RIF low concentrations increased to 118.7%.

The autosampler stability test shows an increase in 25-D-RIF percentage of nominal concentration and a reduction in RIF-Q and 3-F-RIF (all in LQC only) after 12 h. More deterioration occurred in LQC than HQC of 3-F-RIF and RIF-Q (38.7% vs. 83.6%, and 29.7% vs. 63.1%, respectively). Both LQC and HQC levels of 25-D-RIF have increased to more than 122%.

All tested compounds and IS stock solutions were found to be stable for 1 month at −20 °C. We measured 8-month working solution stability and found that RIF-Q, 3-F-RIF, 25-D-RIF, and INH were unstable (Table 3).

Moreover, we evaluated stability for all compounds after 24 h and 30 days in different urine pH ranges from 4 to 8 at −20 °C. We observed that all components were stable at pH 6 during 24 h, and ETH was stable in all pH. However, after 30 days, RIF-Q and 3-F-RIF became significantly unstable in all pH. Other components remained stable at pH 6 to 8, except for 25-D-RIF, which was unstable in alkaline pH, and INH in acidic pH (Table 3).

**Figure 2:**
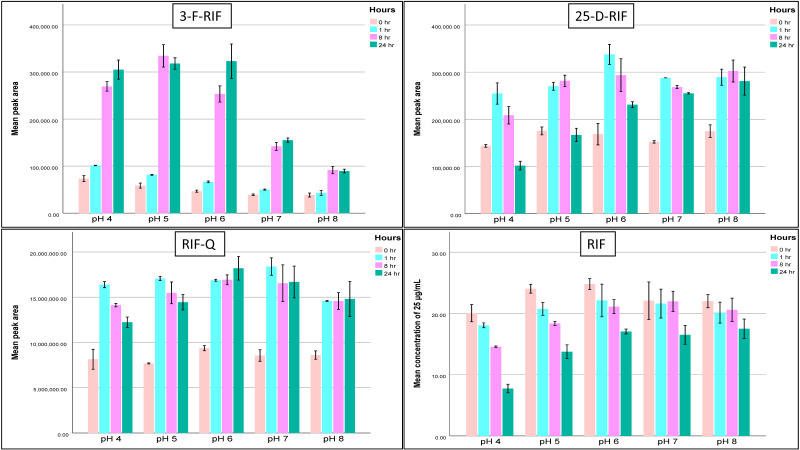
Changes in RIF and its metabolites (RIF-Q, 3-F-RIF, and 25-D-RIF) areas after 1 h, 8 h, and 24 h at 37.5 °C in pH 4 to pH 8. Abbreviations: 25-D-RIF—25-desacetylrifampicin; 3-F-RIF—3-formylrifampicin; RIF-Q—rifampicin quinone; RIF—rifampicin.

**Figure 3:**
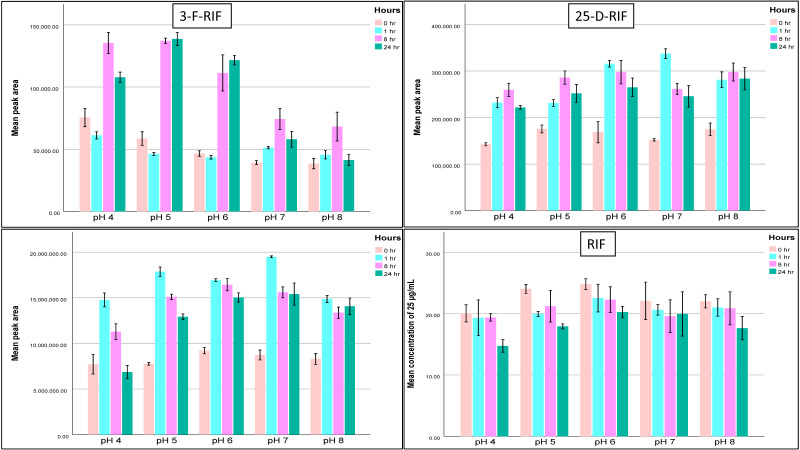
Changes in RIF and its metabolites (RIF-Q, 3-F-RIF, and 25-D-RIF) areas after 1 h, 8 h, and 24 h at 20.5 °C in pH 4 to pH 8. Abbreviations: 25-D-RIF—25-desacetylrifampicin; 3-F-RIF—3-formylrifampicin; RIF-Q—rifampicin quinone; RIF—rifampicin.

Moreover, we extended stability tests for RIF, INH, PZA, and ETH to mimic the collection process at 37.5 °C and room temperature (20.5 °C) (Table 4, Figure 2 and Figure 3). At 37.5 °C, RIF was stable primarily in pH 6 and 7 up to 8 h, INH in pH 6 and 7 up to 24 h, PZA and ETH were stable in pH from 4 to 8 up to 24 h. The results remained almost the same at room temperature.

We also observed an increase in the RIF-Q area throughout stability studies while there was a reduction in RIF concentrations, the process that was much enhanced at 37.5 °C. Also, 25-D-RIF and 3-F-RIF concentrations had the same pattern of RIF (Figure 2).

#### 2.1.7. Dilution Integrity

Dilution integrity was evaluated by analysing spiked urine at analyte concentrations above HQC, and the patient samples. At the dilution factor 10, 25-D-RIF, INH, and ETH in the spiked samples had good results in terms of precision and accuracy with values of 11.4% and 85.4%, 4.3% and 107.6%, and 14.7% and 90.5%, respectively. RIF-Q, 3-F-RIF, and PZA showed lower accuracy with 33.2%, 54.9%, and 66.4%, respectively. It is worth mentioning that ETH also showed good integrity at dilution factor 100. When we compared the concentrations determined in the patient’s urine samples before and after dilution, the results were accurate for all compounds except RIF and RIF-Q. This suggests that RIF and its degradation products should be determined in undiluted urine samples.

#### 2.1.8. Application to Clinical Samples

The established UPLC-MS/MS method was applied to measure urine concentrations of RIF, INH, PZA, ETH, and RIF metabolites in the samples taken from five patients undergoing anti-TB treatment. The determined concentrations of the tested compounds except ETH were within the analytical ranges of the method (Table 5). Hence, for ETH we extended the calibration curve as discussed in Section 2.1.2.

## 3. Discussion

MS conditions have been adjusted to obtain a high sensitivity for the anti-TB drugs and rifampicin degradation products. All of the analytes investigated in this study generated the prominent protonated molecular ions [M + H]^+^ in positive ion mode and these ions were selected as precursor ions for the MS/MS fragmentation analysis of the compounds. The precursor ion of RIF with *m*/*z* 824.3 yielded the most intense product ion of *m*/*z* 792.2 due to loss of CH_3_OH. The second product ion with 398.7 was formed as a result of ring cleavage and separation of aromatic and aliphatic sides of the RIF structure. As RIF-Q is an oxidized form of RIF, its precursor ion and product ions had *m*/*z* similar to RIF, 822.2 and 790.2 and 397.6, respectively (Table 6). To increase the selectivity of the method and avoid interferences, the product ions with *m*/*z* 398.7 for RIF and 790.2 for RIF-Q were chosen for quantitative analysis. The parent ion of 3-F-RIF with *m*/*z* 727.2 undergoes fragmentation primarily by loss of water and -CH_2_CO moieties, resulting in a product ion of 667.1. The mass fragmentation pattern of 25-D-RIF was analogous to that of RIF and RIF-Q. For PZA, the fragmentation *m*/*z* 124.0 to 81.0 was selected, which was the result of the separation of the aromatic ring from the amide group. The precursor ion of INH with *m*/*z* 138.0 yielded the most intense product of 121.0 due to loss of ammonia, while ETH precursor ion of 205.1 forms the fragment with *m*/*z* of 116.1 due to cleavage of ethylenediamine structure (Table 6).

We successfully validated a method according to FDA guidance [38] for urine quantification of INH, PZA, RIF, ETH, 25-D-RIF, 3-F-RIF, and RIF-Q regarding selectivity, linearity and LLOQ, precision and accuracy, matrix effect, carry-over, and stability under different host sample and environmental conditions. Such a method carries the potential application for expanded access to pharmacokinetic testing and TDM in TB endemic settings for clinical trials or operational research.

The sample volume required for analysis (10 µL) and LLOQ values for RIF, 25-D-RIF, and INH are lower than previously reported [27,39]. Panchagnula et al. used the HPLC-UV method with a quantification range of 20–200 μg/mL for RIF and 10–50 μg/mL for 25-D-RIF. However, the method utilized a larger volume of sample (50–300 µL) and involved a multi-step sample preparation before analysis [27]. Similarly, using HPLC-UV, Kumar et al. analyzed INH in the quantification range of 1.25 to 40.0 µg/mL. The authors had to filter the urine before processing and used a 100 µL injection volume [39]. Breda et al. successfully reported an HPLC-FLD analysis of ETH in the quantification range of 10–500 µg/mL in urine; however, its method was based on precolumn derivatization. Also, they used 100 µL of urine with a 200 µL injection volume [26]. Other methods reported lower LLOQ values than ours [29,40]. Hashiguchi et al. reported 0.3 μg/mL of INH LLOQ, but their method was focused on INH and its acetyl metabolite to detect INH adherence for analysis using thin-layer chromatography [40]. Additionally, Mishra et al. reported a quantification range of INH was 0.03–10.0 μg/mL [29]; however, our extended calibration range of 0.5–100 μg/mL allowed us to quantify higher concentrations of INH in patients (average measured concentration was 48.7 ± 22.7 μg/mL) (Table 5). Thus, the current multi-drug process may represent the ideal balance of testing the most commonly prescribed TB drugs over a range of clinically observed quantification.

A recent systematic review by Rao et al. [12] reported on 43 articles on different TB drugs and observed that visual inspection of urine using the Arkansas method was the most common method of testing adherence among patients taking INH. The Arkansas test is a reliable method for monitoring morning INH doses in children 4 h after ingestion. While it is more affordable than HPLC-MS/MS, its sensitivity decreases as INH metabolite concentrations reduce over time. The test is not useful for monitoring INH ingestion 24 h after the dose [41]. While urine colorimetric testing is promising for the measurement of drug exposure and dose adjustment at the point of care [13,14,15] comparatively, LC-MS/MS methods offer simultaneous analysis of multiple TB drugs with high sensitivity and selectivity conducive to pharmacokinetic/pharmacodynamics study for clinical trials among more representative populations, or for testing drug exposure in operational research settings as new drug regimens are rolled out to diverse (nonclinical trial) populations. Additionally, LC-MS/MS requires relatively low sample volumes and involves simple nonselective sample preparation techniques [25]. Hence, it is suitable for analyzing TB drugs in urine.

Up to now, no LC-MS/MS method has been reported for simultaneous analysis of RIF-Q, 3-F-RIF, or PZA in urine. Our method enabled an excellent chromatographic separation of RIF from its degradation products (Figure 1), which may be particularly important given the numerous clinical trials underway for higher-dose rifamycins in different forms of TB disease [42,43,44]. The method accurately quantified all the analytes, including RIF-Q, which other authors observed in clinical samples in plasma [45,46]. However, it is still unclear if RIF-Q plays a role in RIF-induced adverse reactions [47]. Even though we could determine its concentration, 1.9 ± 0.8 µg/mL, it is essential to highlight that the matrix highly influences the concentration (Table 2). Differences in pH of urine samples taken from six subjects likely contributed to the observed variability in matrix effect for RIF-Q, INH, and PZA. As shown in Table 3, the pH of the sample significantly affected the stability of the analytes, with the greatest stability for RIF, PZA, INH, and ETH at pH 5–7.

While working solutions were stable for 1 month at −20 °C [37], an 8-month assessment revealed RIF-Q, 3-F-RIF, 25-D-RIF, and INH instability. Short-term stability data indicated metabolite stability within 24 h when frozen, but 30 days showed significant decomposition of RIF-Q and 3-F-RIF. Most metabolites remained stable at pH 6 to 8, except for 25-D-RIF in alkaline pH and INH in acidic conditions (Table 3).

Our stability assessment at elevated temperature (simulating potential clinical environment during the urine collection process in warmer ambient temperatures) showed that RIF was more stable (up to 8 h) at a pH ranging from 6 to 7. INH was stable in pH 6 and pH 7 up to 24 h. PZA and ETH were stable in pH from 4 to pH 8 up to 24 h. These data suggest that maintaining the pH of collected urine in the range of 6 to 7 during drug monitoring studies for up to 8 h should provide a reliable sample (Table 4). Hence, adding a buffer to maintain a desired pH during urine collection in clinical settings should be further evaluated.

Analysis of the tested compounds using a validated UPLC-MS/MS method in urine samples from five patients showed that the samples contained RIF-Q and 3-F-RIF (Table 5), suggesting that RIF may be degraded during sample preparation or storage. The concentration of RIF-Q was in the range of 0.71–2.62 µg/mL (average 1.9 ± 0.8 µg/mL), and comprised 3.6–13.2% of the sum of RIF and RIF-Q (almost twice than reported in plasma [37]). Similarly, 3-F-RIF peak was detected in all samples (Figure 1), and the concentration of the compound was an average of 1.3 ± 1.3 µg/mL (Table 5), while it was previously below LLOQ in plasma [37]. Given the potential conversion between RIF and RIF-Q and the variations in pH during urine storage, additional research is needed to explore the degradation pathway of RIF.

## 4. Materials and Methods

### 4.1. Standards and Reagents

High-quality reference standards were sourced to ensure the precision and accuracy of our analytical methodology. PZA as a certified reference material, INH (≥99% purity), and RIF (>97% purity) were procured from Sigma-Aldrich (St. Louis, MO, USA). In addition, the following reference standards and internal standards were obtained from Toronto Research Chemicals (North York, ON, Canada): ETH (98% purity), 25-D-RIF (95.06% purity), RIF-Q (95.19% purity), and 3-F-RIF (96.42% purity); ethambutol-D4 (ETH-IS; 98% purity), rifampicin-D3 (RIF-IS; 98.15% purity), pyrazinamide-15N,D3 (PZA-IS; 99.5% purity). Acetonitrile, water, and methanol were procured from Sigma-Aldrich (St. Louis, MO, USA), while isopropanol was sourced from Fisher Chemical (Fair Lawn, NJ, USA). All solvents met LCMS grade specifications. Formic acid (FA) (>99.0% purity) was supplied by Fisher Chemical. Ammonium formate (≥99% purity) was obtained from Honeywell (Morristown, NJ, USA). Blank urine of individual donors for method validation was received from BioIVT (Westbury, NY, USA).

### 4.2. Instrumentation and Chromatographic Conditions

An ExionLC AD system (AB Sciex, Framingham, MA, USA) featuring a Kinetex Polar C18 column (2.6 µm; 150 × 3 mm), safeguarded by a UHPLC C18 (3.0 mm ID) guard column (Phenomenex, Torrance, CA, USA) was used for chromatographic separation. The column oven and the autosampler were maintained to ensure optimal performance at 40 °C and 15 °C, respectively. The volume of injection was 4 µL.

The mobile phase was a mixture of two components: Mobile Phase A (MPA) contained 5 mM ammonium formate in water with 0.1% FA, and Mobile Phase B (MPB) consisted of acetonitrile with 0.1% FA. The mobile phase was delivered at a flow rate of 0.35 mL/min and followed this gradient profile: initially, at 0 min, it was 99% MPA and 1% MPB (*v*/*v*). After 8 min, it shifted to 1% MPA and 99% MPB and remained so until the 10-min mark. At 10.5 min, it returned to 99% MPA, at which it stayed until the end of the run at 12 min.

For rinsing, a mixture of deionized MilliQ water (Millipore, Billerica, MA, USA) and acetonitrile in a 50:50 (*v*/*v*) ratio, with 0.1% FA, was used both externally and internally to clean the needle. To prevent mass spectrometer contamination, the mobile phase was directed to waste from 0.1 to 1.0 min and again after 9.5 min into the run.

To address the significant carry-over of INH and PZA, an additional cleaning method was implemented for both the autosampler and column. We injected a mixture of isopropanol and water (50:50, *v*/*v*; 4 µL) twice after 4–5 samples. The composition of MPA and MPB remained the same but was delivered using a W-shaped gradient profile at a constant flow rate of 0.35 mL/min. The gradient profile was as follows: 0 min—99:1 (MPA: MPB, *v*/*v*), 1 min—99:1, 2 min—1:99, 3 min—1:99, 4 min—99:1, 5 min—1:99, 6 min—1:99, 7 min—99:1, 8 min—1:99, 9 min—1:99, 10 min—99:1, 12 min—99:1.

### 4.3. Mass Spectrometer Settings

Detection was carried out using an AB Sciex QTRAP 6500+ mass spectrometer (Framingham, MA, USA) equipped with an IonDrive Turbo V Source. It operated in positive ionization mode (ESI+) employing multiple reaction monitoring modes (MRMs). The specific ion source parameters utilized were as follows: the source temperature was set at 400 °C, the ion spray voltage was maintained at 5 kV, and the collision gas, nitrogen, was set to a medium-level setting. The pressures for ion source gas 1 and 2 (zero air) were 60 psi and 40 psi, respectively, while the curtain gas, also nitrogen, was set at 30 psi. The nitrogen and zero air necessary for these operations were supplied by a Genius 1024 generator from Peak Scientific (Billerica, MA, USA). For each transition being monitored, a dwell time of 150 ms was employed to ensure accurate and precise detection. Details regarding the monitored transitions and compound-specific parameters can be found in Table 6.

### 4.4. Stock and Standard Solutions

Stock solutions of 25-D-RIF, RIF-Q, ETH-IS, and PZA-IS were prepared with concentrations of 1 mg/mL. ETH, INH, RIF, and PZA stock solutions were prepared at 10 mg/mL. Additionally, 3-F-RIF was prepared at a concentration of 2.5 mg/mL, and RIF-IS was prepared at 0.5 mg/mL. These stock solutions were created by dissolving the appropriate amount of each compound in methanol, except for INH, which was dissolved in water.

To prepare the standard solutions, the stock solutions were diluted with methanol to achieve the desired concentrations as follows:

- RIF, ETH, INH, and PZA: 5.0, 10.0, 25.0, 50.0, 100.0, 250.0, 500.0, and 1000.0 µg/mL.
- 25-D-RIF, RIF-Q, and 3-F-RIF: 1.0, 2.5, 5.0, 10.0, 25.0, 50.0, 100.0, and 200.0 µg/mL.
- ETH-IS and RIF-IS: 0.5 µg/mL.
- PZA-IS: 5 µg/mL.

All of these solutions were prepared in amber glass vials and stored at a temperature of −20 °C to maintain their stability and prevent degradation. ETH, INH, and PZA were prepared as a mixture (MIX I), 25-D-RIF, RIF-Q, and 3-F-RIF as MIX II, and RIF solution was prepared separately.

### 4.5. Preparation of Calibrators, Quality Control Samples and Biological Samples

The standard solutions of RIF, and compounds in MIX I and MIX II were further diluted 10-fold (5 µL standard solution MIX I + 5 µL standard solution MIX II + 40 µL blank urine; for RIF: 5 µL standard solution RIF + 45 µL blank urine). Final calibrators were at the following concentrations:

- RIF, ETH, INH, and PZA: 0.5, 1.0, 2.5, 5.0, 10.0, 25.0, 50.0, and 100.0 µg/mL.
- 25-D-RIF, RIF-Q, and 3-F-RIF: 0.1, 0.25, 0.5, 1.0, 2.5, 5.0, 10.0, and 20.0 µg/mL.

It is worth noting that two distinct calibration samples were prepared as we observed that RIF-Q was present in small concentrations in the RIF standard solutions (freshly prepared). Hence, the first calibration sample exclusively contained RIF, while the second included a blend of all the other analytes. This approach ensured the accuracy and precision of the calibration.

In addition, QC samples were prepared at various concentration levels, including the LLOQ, low, medium, and high concentrations, as outlined in Table 1. These QC samples served for evaluating the performance and reliability of the analytical method.

At the next stage, a 10 µL aliquot of urine calibrators and QC samples were combined with 100 µL of a freshly prepared internal standards (ETH-IS, RIF-IS, and PZA-IS) solution in cold methanol (5 °C). A similar procedure was implemented for biological samples (10 µL urine + 100 µL of internal standards). Next, this mixture was vigorously shaken for 10 min and subsequently subjected to centrifugation at 15,700× *g* for 10 min, maintaining a temperature of 5 °C.

Following centrifugation, a 20 µL portion of the supernatant was carefully transferred to a glass vial, where it was then mixed with 100 µL of cold acetonitrile containing 0.1% FA at 5 °C. This mixture was vortexed to ensure thorough mixing.

To enhance sample stability, the samples were kept at a temperature of 5 °C until they were ready to be transferred to the autosampler for analysis.

### 4.6. Validation

The method was validated according to the U.S. Food and Drug Administration (FDA) guidance [38].

#### 4.6.1. Selectivity

The chromatograms of blank urine samples were carefully examined from six individuals to assess method selectivity. These blank urine samples were spiked with IS and analytes at an LLOQ concentration. This analysis aimed to identify any potential interferences that might occur at specific retention times during the chromatographic process.

#### 4.6.2. Calibration Curves

Calibration curves were constructed by plotting the ratio of the peak area of the analyte to that of IS against the analyte concentration. These curves covered a concentration range of 0.5 to 100.0 µg/mL for ETH, INH, RIF, and PZA, and from 0.1 to 20.0 µg/mL for 25-D-RIF, RIF-Q, and 3-F-RIF. The selection of internal standards was as follows: RIF-IS served as the internal standard for RIF and its metabolites: 25-D-RIF, RIF-Q, 3-F-RIF; PZA-IS was used for INH and PZA, and ETH-IS was employed for ETH. For each calibration curve, both linear (with weighting factors of 1/x or 1/x^2^) and nonlinear regression methods (using a power model with no specific weighting) were applied. These approaches allowed for a comprehensive evaluation of the calibration data to ensure accuracy and reliability in the quantification of the analytes.

#### 4.6.3. LLOQ, Precision, and Accuracy

LLOQ was the lowest concentration of the analytes measured by the method with relative error (RE) and relative standard deviation (RSD) ≤ 20%. RIF-Q, 3-F-RIF, and 25-D-RIF had LLOQ of 0.1 µg/mL in urine, and RIF, INH, PZA, and ETH—0.5 µg/mL (Table 1).

Intra- and inter-day accuracy and precision were assessed in five replicates by determining QCs on the same day and over 5 consecutive days (Table 1). The RSD between the nominal and the measured concentrations should be within ±15% for QCs and ±20% for the LLOQ.

#### 4.6.4. Carry-Over and Dilution Integrity

To evaluate carry-over, a blank sample was injected just after HQC. It was considered acceptable if the blank sample’s signal was ≤20% of the LLOQ’s sample signal.

The dilution integrity was analyzed in urine samples containing the tested compounds at concentrations above the calibration curve (RIF-Q, 3-F-RIF, and 25-D-RIF—30 µg/mL; RIF—300 µg/mL; INH, PZA, and ETH—200 µg/mL). The samples were prepared in five replicates and diluted ten times to be measured within the calibration curve range (RIF-Q, 3-F-RIF, and 25-D-RIF—3 µg/mL; RIF—30 µg/mL; INH, PZA, and ETH—20 µg/mL). For ETH, a 100 times dilution was also evaluated. Additionally, the effect of dilution was tested on patient urine samples. The dilution of the samples should not affect the accuracy and precision (RSD ≤ 15%, accuracy 85–115%).

#### 4.6.5. Matrix Effect

We prepared two series to investigate the matrix effect. The first series (series I) was the matrix (urine from 6 different people) spiked with QCs at low and high concentrations of the analytes and processed using the protocol described in Section 4.5. The second series (series II) was prepared using water spiked with analytes at equivalent concentrations. For each equivalent concentration, MF was determined by dividing the peak area from Series I by the peak area in Series II. To obtain the IS normalized MF, the MF for the analyte was divided by the MF for the IS, and its RSD should be ≤15%.

#### 4.6.6. Stability

We assessed the stability of the tested compounds in urine samples under various storage conditions, including short- and long-term stability at −20 °C, short-term stability at room temperature, stability in urine samples with different pH levels from 4 to 8, and stability in processed samples in an autosampler (15 °C). The samples were prepared at low and high QC concentration levels of the analytes in three replicates. Additionally, the stability of stock and working solutions was investigated under specific temperature conditions. The stability of specific substances (RIF, INH, PZA, and ETH) in urine was also examined under different pH levels and at room temperature (20.5 °C) and 37.5 °C settings for varying time intervals (1 h, 8 h, and 24 h). Before spiking with analytes, urine pH was adjusted by adding 1M HCl or 1M NaOH.

#### 4.6.7. Application to Clinical Samples

The validated analytical method was employed for the quantification of RIF, RIF-Q, 25-D-RIF, INH, PZA, and ETH in urine specimens obtained from a cohort of five adult individuals receiving treatment for active TB disease through a first-line regimen containing RIF, INH, PZA, and ETH. These study participants were recruited from a range of healthcare institutions, encompassing those in New Jersey, such as the Rutgers Global TB Institute’s Lattimore Practice, and clinics in Middlesex, Hudson, and Patterson County. Additionally, participants were recruited from healthcare facilities in Virginia, including the University of Virginia and adjacent Virginia Department of Health clinics. The eligibility criteria for participation in the study stipulated the exclusion of individuals with conditions such as incontinence, anuria, pregnancy, or breastfeeding. Notably, all study participants provided informed consent prior to their inclusion in the research endeavor. Urine samples were collected continuously over 4 h following administration of the drugs [18]. Samples were prepared as described in Section 4.5. Human subjects’ approval was obtained through Rutgers Health Sciences IRB Pro2018001857 and University of Virginia Health Sciences IRB HSR #20944.

## 5. Conclusions

We have successfully developed and validated the first LC-MS/MS method for simultaneous analysis of INH, PZA, RIF, and ETH in urine samples to facilitate TDM of primary first-line anti-TB agents. Additionally, we performed the extended stability quantification for study in clinical trials, operational research, or moving closer to the point of care for TDM and dose adjustment in patients treated for TB in a diversity of settings, including the quantification of RIF’s metabolites/degradation products, 25-D-RIF, 3-F-RIF, and RIF-Q. The study proved that pH of urine samples and temperature significantly affected the analyte stability. Further recommendations are needed regarding processing the urine sample pH after collection. In particular, maintaining the pH of collected urine in the range of 6 to 7 during drug monitoring studies for up to 8 h will increase the stability of RIF.

## Figures and Tables

**Table 1:** Intra- and inter-day precision and accuracy for anti-TB drugs and RIF metabolism/degradation products in urine.

Compound	QC Level	Conc. (µg/mL)	Precision (%RSD)		Accuracy (%)	
			Intra-Day	Inter-Day	Intra-Day	Inter-Day
			n = 5	n = 5	n = 5	n = 5
RIF	LLOQ	0.5	19.4	4.7	105.9	97.3
	Low	1	11.3	8.7	108.0	91.8
	Medium	10	13.2	7.2	98.5	93.0
	High	100	10.0	11.9	99.3	111.7
RIF-Q	LLOQ	0.1	15.5	12.9	115.7	99.4
	Low	0.25	4.2	9.4	104.9	91.0
	Medium	2.5	9.2	9.2	93.2	94.5
	High	20	6.9	4.3	107.4	114.0
3-F-RIF	LLOQ	0.1	8.9	10.5	110.5	102.2
	Low	0.25	7.2	11.1	108.6	102.0
	Medium	2.5	13.3	8.8	87.7	90.0
	High	20	8.3	8.9	108.6	95.8
25-D-RIF	LLOQ	0.1	6.2	5.0	94.3	106.2
	Low	0.25	12.3	6.7	101.7	99.9
	Medium	2.5	9.7	6.0	101.9	104.3
	High	20	7.4	4.2	99.6	107.8
INH	LLOQ	0.5	8.2	6.7	98.1	86.4
	Low	1	10.0	8.8	104.3	97.0
	Medium	10	5.5	4.5	98.8	93.8
	High	100	3.9	2.5	107.8	100.1
PZA	LLOQ	0.5	18.1	8.3	95.7	87.4
	Low	1	6.0	8.5	101.5	103.3
	Medium	10	6.5	1.4	97.6	104.2
	High	100	4.3	4.5	101.7	109.0
ETH	LLOQ	0.5	16.3	11.3	98.6	96.2
	Low	1	7.2	8.8	107.9	108.9
	Medium	10	9.4	14.3	91.2	106.2
	High	100	3.9	7.1	104.7	109.1

Abbreviations: 25-D-RIF—25-desacetylrifampicin; 3-F-RIF—3-formylrifampicin; ETH—ethambutol; INH—isoniazid; PZA—pyrazinamide; RIF-Q—rifampicin quinone; RIF—rifampicin.

**Table 2:** IS-normalized matrix factor for the analytes at the low and high QC levels.

Compound	QC Level	Conc. [mg/L]	IS-Normalized MF (n = 6)		%RSD
			Mean	SD	
RIF	Low	1	1.27	0.06	5.0
	High	100	1.06	0.14	13.1
RIF-Q	Low	0.25	0.43	0.19	45.2
	High	20	0.62	0.32	51.9
3-F-RIF	Low	0.25	0.51	0.06	11.1
	High	20	0.66	0.10	14.5
25-D-RIF	Low	0.25	1.74	0.16	9.2
	High	20	1.55	0.20	12.6
INH	Low	1	0.37	0.05	12.6
	High	100	0.45	0.08	18.3
PZA	Low	1	1.16	0.19	16.1
	High	100	0.70	0.15	21.7
ETH	Low	1	0.85	0.11	12.8
	High	100	0.83	0.06	7.2

Abbreviations: 25-D-RIF—25-desacetylrifampicin; 3-F-RIF—3-formylrifampicin; ETH—ethambutol; INH—isoniazid; PZA—pyrazinamide; RIF-Q—rifampicin quinone; RIF—rifampicin.

**Table 3:** Stability of the analytes in urine samples (expressed by percentage of nominal concentration, %).

Compound	QC Level	Bench-Top Stability at RT		Autosampler (12 h, 15 °C)	Autosampler (24 h, 15 °C)	Working Solution Stability (−20 °C)		Short-Term Stability (24 h, −20 °C)					Long-Term Stability (30 Days, −20 °C)				
		1 h	4 h			1 Month	8 Months	pH 4	pH 5	pH 6	pH 7	pH 8	pH 4	pH 5	pH 6	pH 7	pH 8
RIF	Low	98	90	90	92	103	95	74	81	87	86	90	83	105	115	110	101
	High	101	96	99	100	101	94	85	87	90	88	89	96	103	100	95	100
RIF-Q	Low	69	39	84	39	101	127	80	81	92	76	90	55	55	64	54	46
	High	87	79	92	96	98	88	119	97	98	119	126	55	27	29	50	80
3-F-RIF	Low	97	61	63	30	97	61	60	58	54	50	64	24	39	70	83	77
	High	91	88	97	92	100	78	119	97	98	119	126	11	20	84	116	104
25-D-RIF	Low	111	119	120	123	104	182	102	112	120	117	114	92	90	87	101	98
	High	95	111	106	123	102	168	103	112	108	115	107	90	110	110	142	137
INH	Low	99	99	98	91	110	124	75	92	96	97	85	44	47	85	86	77
	High	94	94	102	100	109	115	93	103	107	114	105	88	96	98	114	95
PZA	Low	111	104	105	98	105	107	98	90	94	100	79	102	95	102	110	91
	High	102	106	109	109	99	112	106	103	112	110	133	88	78	87	94	111
ETH	Low	112	108	115	114	104	-	111	108	113	114	102	105	107	105	104	101
	High	112	114	111	105	98	-	110	104	108	97	104	99	111	101	114	103

Abbreviations: 25-D-RIF—25-desacetylrifampicin; 3-F-RIF—3-formylrifampicin; ETH—ethambutol; INH—isoniazid; PZA—pyrazinamide; RIF-Q—rifampicin quinone; RIF—rifampicin.

**Table 4:** Stability of TB drugs in urine stored at 37.5 °C and at 20.5 °C for 1–24 h to mimic the urine collection process in working conditions in various TB endemic settings. Values were expressed by percentage of nominal concentration (%).

Compound	Initial Conc. [mg/L]	1 h					8 h					24 h				
		pH 4	pH 5	pH 6	pH 7	pH 8	pH 4	pH 5	pH 6	pH 7	pH 8	pH 4	pH 5	pH 6	pH 7	pH 8
After incubation at 37.5 °C																
RIF	25	82	94	96	88	88	57	75	82	86	80	30	55	66	67	68
INH	50	88	89	92	90	87	79	84	92	94	81	79	78	86	86	76
PZA	50	86	90	93	90	88	92	92	93	94	86	92	90	91	89	86
ETH	50	88	85	92	100	90	89	93	94	87	99	89	97	98	92	92
After incubation at 20.5 °C																
RIF	25	79	87	99	89	87	76	82	86	81	81	58	71	78	77	71
INH	50	86	86	90	91	87	79	77	92	88	91	79	74	87	90	79
PZA	50	95	101	99	103	103	90	85	88	87	85	92	87	85	93	85
ETH	50	86	98	87	98	99	97	87	87	92	94	91	88	96	104	91

Abbreviations: RIF—rifampicin; INH—isoniazid; PZA—pyrazinamide; ETH—ethambutol.

**Table 5:** Mean urine concentrations of anti-TB drugs and RIF metabolites in patients’ samples collected over 4 h after administration (n = 5).

Compound	Conc. (µg/mL) (Mean ± SD)
RIF	17.9 ± 7.8
RIF-Q	1.9 ± 0.8
3-F-RIF	1.3 ± 1.3
25-D-RIF	4.1 ± 2.5
INH	48.7 ± 22.7
PZA	54.3 ± 19.5
ETH	391.7 ± 200.3

Abbreviations: 25-D-RIF—25-desacetylrifampicin; 3-F-RIF—3-formylrifampicin; ETH—ethambutol; INH—isoniazid; PZA—pyrazinamide; RIF-Q—rifampicin quinone; RIF—rifampicin.

**Table 6:** Optimized mass spectrometric parameters used for detection of the analytes and IS in the MRM mode. Quantification transitions are highlighted in bold.

Analyte	Precursor Ion (*m*/*z*)	Fragment Ion (*m*/*z*)	Collision Energy (V)
RIF	824.3	792.2	25
		**398.7**	37
RIF-Q	822.2	**790.2**	25
		397.6	37
3-F-RIF	727.2	**667.1**	17
		641.2	71
25-D-RIF	782.3	**750.2**	17
		399.7	33
INH	138.0	**121.0**	19
		79.0	39
PZA	124.0	64.0	7
		**81.0**	23
ETH	205.1	**116.1**	21
		145	11
RIF-IS	827.3	795.3	25
		**151.1**	37
PZA-IS	128.0	**84.0**	25
		99.9	9
ETH-IS	209.2	**120.0**	21
		149.2	9

Abbreviations: 25-D-RIF—25-desacetylrifampicin; 3-F-RIF—3-formylrifampicin; ETH—ethambutol; ETH-IS—ethambutol-D4; INH—isoniazid; PZA—pyrazinamide; PZA-IS—pyrazinamide-15N,D3; RIF-Q—rifampicin quinone; RIF—rifampicin; RIF-IS—rifampicin-D3.

## Data Availability

The data presented in this study are available on request from the corresponding author. The data are not publicly available due to privacy restrictions.

## References

[1] Global Tuberculosis Programme (2020). Global Tuberculosis Report 2022.

[2] Tuberculosis (TB)—Treatment for TB Disease. https://www.cdc.gov/tb/topic/treatment/tbdisease.htm.

[3] Alsultan A., Peloquin C.A. (2014). Therapeutic Drug Monitoring in the Treatment of Tuberculosis: An Update. Drugs.

[4] Peloquin C.A. (2002). Therapeutic Drug Monitoring in the Treatment of Tuberculosis. Drugs.

[5] Zuur M.A., Bolhuis M.S., Anthony R., den Hertog A., van der Laan T., Wilffert B., de Lange W., van Soolingen D., Alffenaar J.-W.C. (2016). Current Status and Opportunities for Therapeutic Drug Monitoring in the Treatment of Tuberculosis. Expert Opin. Drug Metab. Toxicol..

[6] Choi R., Jeong B.-H., Koh W.-J., Lee S.-Y. (2017). Recommendations for Optimizing Tuberculosis Treatment: Therapeutic Drug Monitoring, Pharmacogenetics, and Nutritional Status Considerations. Ann. Lab. Med..

[7] Weiner M., Peloquin C., Burman W., Luo C.-C., Engle M., Prihoda T.J., Mac Kenzie W.R., Bliven-Sizemore E., Johnson J.L., Vernon A. (2010). Effects of Tuberculosis, Race, and Human Gene SLCO1B1 Polymorphisms on Rifampin Concentrations. Antimicrob. Agents Chemother..

[8] Azuma J., Ohno M., Kubota R., Yokota S., Nagai T., Tsuyuguchi K., Okuda Y., Takashima T., Kamimura S., Fujio Y. (2013). NAT2 Genotype Guided Regimen Reduces Isoniazid-Induced Liver Injury and Early Treatment Failure in the 6-Month Four-Drug Standard Treatment of Tuberculosis: A Randomized Controlled Trial for Pharmacogenetics-Based Therapy. Eur. J. Clin. Pharmacol..

[9] Dekkers B.G.J., Akkerman O.W., Alffenaar J.W.C. (2019). Role of Therapeutic Drug Monitoring in Treatment Optimization in Tuberculosis and Diabetes Mellitus Comorbidity. Antimicrob. Agents Chemother..

[10] Chang M.J., Chae J.-W., Yun H.-Y., Lee J.I., Choi H.D., Kim J., Park J.S., Cho Y.-J., Yoon H.I., Lee C.-T. (2015). Effects of Type 2 Diabetes Mellitus on the Population Pharmacokinetics of Rifampin in Tuberculosis Patients. Tuberc. Edinb. Scotl..

[11] Märtson A.-G., Burch G., Ghimire S., Alffenaar J.-W.C., Peloquin C.A. (2021). Therapeutic Drug Monitoring in Patients with Tuberculosis and Concurrent Medical Problems. Expert Opin. Drug Metab. Toxicol..

[12] Rao P.S., Modi N., Nguyen N.-T.T., Vu D.H., Xie Y.L., Gandhi M., Gerona R., Metcalfe J., Heysell S.K., Alffenaar J.-W.C. (2023). Alternative Methods for Therapeutic Drug Monitoring and Dose Adjustment of Tuberculosis Treatment in Clinical Settings: A Systematic Review. Clin. Pharmacokinet..

[13] Rao S.P., Reed K., Modi N., Handler D., de Guex K.P., Yu S., Kagan L., Reiss R., Narayanan N., Peloquuin C.A. (2023). Isoniazid Urine Spectrophotometry for Prediction of Serum Pharmacokinetics in Adults with Tuberculosis. Int. J. Tuberc. Lung Dis. Off. J. Int. Union Tuberc. Lung Dis..

[14] Thomas T.A., Lukumay S., Yu S., Rao P., Siemiątkowska A., Kagan L., Augustino D., Mejan P., Mosha R., Handler D. (2023). Rifampin Urinary Excretion to Predict Serum Targets in Children with Tuberculosis: A Prospective Diagnostic Accuracy Study. Arch. Dis. Child..

[15] Xie Y.L., Modi N., Handler D., Yu S., Rao P., Kagan L., Petros de Guex K., Reiss R., Siemiątkowska A., Narang A. (2023). Simplified Urine-Based Method to Detect Rifampin Underexposure in Adults with Tuberculosis: A Prospective Diagnostic Accuracy Study. Antimicrob. Agents Chemother..

[16] Meissner P.E., Musoke P., Okwera A., Bunn J.E.G., Coulter J.B.S. (2002). The Value of Urine Testing for Verifying Adherence to Anti-Tuberculosis Chemotherapy in Children and Adults in Uganda. Int. J. Tuberc. Lung Dis..

[17] Zentner I., Modongo C., Zetola N.M., Pasipanodya J.G., Srivastava S., Heysell S.K., Mpagama S., Schlect H.P., Gumbo T., Bisson G.P. (2018). Urine Colorimetry for Therapeutic Drug Monitoring of Pyrazinamide during Tuberculosis Treatment. Int. J. Infect. Dis..

[18] Palanduz A., Gültekin D., Erdem E., Kayaalp N. (2003). Low Level of Compliance with Tuberculosis Treatment in Children: Monitoring by Urine Tests. Ann. Trop. Paediatr..

[19] Ji A.J., Jiang Z., Livson Y., Davis J.A., Chu J.X., Weng N. (2010). Challenges in Urine Bioanalytical Assays: Overcoming Nonspecific Binding. Bioanalysis.

[20] Zhang Z., Liu J., Cheng Y., Chen J., Zhao H., Ren X. (2022). Urine Analysis Has a Very Broad Prospect in the Future. Front. Anal. Sci..

[21] Burman W.J., Gallicano K., Peloquin C. (2001). Comparative Pharmacokinetics and Pharmacodynamics of the Rifamycin Antibacterials. Clin. Pharmacokinet..

[22] Thummel K.E., Shen D.D., Isoherranen N., Brunton L.L., Chabner B.A., Knollmann B.C. (2015). Design and Optimization of Dosage Regimens: Pharmacokinetic Data. Goodman & Gilman’s: The Pharmacological Basis of Therapeutics.

[23] Lacroix C., Hoang T.P., Nouveau J., Guyonnaud C., Laine G., Duwoos H., Lafont O. (1989). Pharmacokinetics of Pyrazinamide and Its Metabolites in Healthy Subjects. Eur. J. Clin. Pharmacol..

[24] Holdiness M.R. (1984). Clinical Pharmacokinetics of the Antituberculosis Drugs. Clin. Pharmacokinet..

[25] Kuhlin J., Sturkenboom M.G.G., Ghimire S., Margineanu I., van den Elsen S.H.J., Simbar N., Akkerman O.W., Jongedijk E.M., Koster R.A., Bruchfeld J. (2019). Mass Spectrometry for Therapeutic Drug Monitoring of Anti-Tuberculosis Drugs. Clin. Mass Spectrom..

[26] Breda M., Marrari P., Pianezzola E., Strolin Benedetti M. (1996). Determination of Ethambutol in Human Plasma and Urine by High-Performance Liquid Chromatography with Fluorescence Detection. J. Chromatogr. A.

[27] Panchagnula R., Sood A., Sharda N., Kaur K., Kaul C.L. (1999). Determination of Rifampicin and Its Main Metabolite in Plasma and Urine in Presence of Pyrazinamide and Isoniazid by HPLC Method. J. Pharm. Biomed. Anal..

[28] Weber A., Opheim K.E., Smith A.L., Wong K. (1983). High-Pressure Liquid Chromatographic Quantitation of Rifampin and Its Two Major Metabolites in Urine and Serum. Rev. Infect. Dis..

[29] Mishra P., Albiol-Chiva J., Bose D., Durgbanshi A., Peris-Vicente J., Carda-Broch S., Esteve-Romero J. (2018). Optimization and Validation of a Chromatographic Method for the Quantification of Isoniazid in Urine of Tuberculosis Patients According to the European Medicines Agency Guideline. Antibiotics.

[30] Mishra P., Pawar R.-P., Bose D., Durgbanshi A., Albiol-Chiva J., Peris-Vicente J., Esteve-Romero J., Jain A. (2019). Stability Studies of Rifampicin in Plasma and Urine of Tuberculosis Patients According to the European Medicines Agency Guidelines. Bioanalysis.

[31] Sutradhar I., Zaman M.H. (2021). Evaluation of the Effect of Temperature on the Stability and Antimicrobial Activity of Rifampicin Quinone. J. Pharm. Biomed. Anal..

[32] Prasad B., Singh S. (2009). In Vitro and in Vivo Investigation of Metabolic Fate of Rifampicin Using an Optimized Sample Preparation Approach and Modern Tools of Liquid Chromatography–Mass Spectrometry. J. Pharm. Biomed. Anal..

[33] Mwila C., Walker R.B. (2020). Improved Stability of Rifampicin in the Presence of Gastric-Resistant Isoniazid Microspheres in Acidic Media. Pharmaceutics.

[34] Shishoo C.J., Shah S.A., Rathod I.S., Savale S.S., Kotecha J.S., Shah P.B. (1999). Stability of Rifampicin in Dissolution Medium in Presence of Isoniazid. Int. J. Pharm..

[35] Abulfathi A.A., Decloedt E.H., Svensson E.M., Diacon A.H., Donald P., Reuter H. (2019). Clinical Pharmacokinetics and Pharmacodynamics of Rifampicin in Human Tuberculosis. Clin. Pharmacokinet..

[36] Singh S., Mariappan T.T., Shankar R., Sarda N., Singh B. (2001). A Critical Review of the Probable Reasons for the Poor Variable Bioavailability of Rifampicin from Anti-Tubercular Fixed-Dose Combination (FDC) Products, and the Likely Solutions to the Problem. Int. J. Pharm..

[37] Karaźniewicz-Łada M., Kosicka-Noworzyń K., Rao P., Modi N., Xie Y.L., Heysell S.K., Kagan L. (2023). New Approach to Rifampicin Stability and First-Line Anti-Tubercular Drug Pharmacokinetics by UPLC-MS/MS. J. Pharm. Biomed. Anal..

[38] (2018). U.S. Food and Drug Administration Bioanalytical Method Validation. Guidance for Industry.

[39] Hemanth Kumar A.K., Sudha V. (2014). Geetha Ramachandran Simple and Rapid Method for Simultaneous Determination of Isoniazid and Acetyl Isoniazid in Urine by HPLC. Asian J. Biomed. Pharm. Sci..

[40] Hashiguchi M., Ohno K., Sakuma A., Hino F., Tanaka T., Ohtsuji M., Matsumoto N., Yanase K., Urae A., Hosogai Y. (2002). A Simplified Method for Detecting Isoniazid Compliance in Patients Receiving Antituberculosis Chemotherapy. J. Clin. Pharmacol..

[41] Amlabu V., Mulligan C., Jele N., Evans A., Gray D., Zar H.J., McIlleron H., Smith P. (2014). Isoniazid/Acetylisoniazid Urine Concentrations: Markers of Adherence to Isoniazid Preventive Therapy in Children. Int. J. Tuberc. Lung Dis..

[42] Jindani A., Atwine D., Grint D., Bah B., Adams J., Ticona E.R., Shrestha B., Agizew T., Hamid S., Jamil B. (2023). Four-Month High-Dose Rifampicin Regimens for Pulmonary Tuberculosis. NEJM Evid..

[43] Espinosa-Pereiro J., Ghimire S., Sturkenboom M.G.G., Alffenaar J.-W.C., Tavares M., Aguirre S., Battaglia A., Molinas G., Tórtola T., Akkerman O.W. (2022). Safety of Rifampicin at High Dose for Difficult-to-Treat Tuberculosis: Protocol for RIAlta Phase 2b/c Trial. Pharmaceutics.

[44] Fregonese F., Apriani L., Barss L., Benedetti A., Cook V., Fisher D., Fox G.J., Johnston J., Long R., Nguyen T.A. (2023). High Dose Rifampin for 2 Months vs Standard Dose Rifampin for 4 Months, to Treat TB Infection: Protocol of a 3-Arm Randomized Trial (2R2). PLoS ONE.

[45] Pršo K., Žideková N., Porvazník I., Solovič I., Mokrý J., Kertys M. (2023). A High-Throughput LC–MS/MS Method for Simultaneous Determination of Isoniazid, Ethambutol and Pyrazinamide in Human Plasma. Rapid Commun. Mass Spectrom..

[46] Kivrane A., Grinberga S., Sevostjanovs E., Igumnova V., Pole I., Viksna A., Bandere D., Krams A., Cirule A., Pugovics O. (2021). LC-MS/MS Method for Simultaneous Quantification of the First-Line Anti-Tuberculosis Drugs and Six Primary Metabolites in Patient Plasma: Implications for Therapeutic Drug Monitoring. J. Chromatogr. B.

[47] Shi F., Li X., Pan H., Ding L. (2017). NQO1 and CYP450 Reductase Decrease the Systemic Exposure of Rifampicin-Quinone and Mediate Its Redox Cycle in Rats. J. Pharm. Biomed. Anal..

